# A Guide to the Practical Examination of the Urine

**Published:** 1879-04

**Authors:** 


*A Guide to the Practical Examination of Urine for the use of Physicians and Students.* By JAMES TYSON, M. D., Professor of General Pathology and Morbid Anatomy in the University of Pennsylvania, one of the Physicians to the Philadelphia Hospital, Fellow of the College of Physicians., etc., etc. Second edition, revised and improved, with illustrations. Philadelphia: Lindsay and Blakiston. 1878. Buffalo: T. H. Butler. Price $1.25.

In the present edition of this little work the author has carefully corrected the previous one, as well as improved it, by incorporating some of the most recent analyses without increasing the size of the work, and has presented the profession a work which is a guide to the practitioner in his daily practice. Its concise form and practical ideas can not fail to insure its success as a general text book upon the examination of urine and its adoption by the profession at large.

